# Vitamin D Deficiency Cause Gender Specific Alterations of Renal Arterial Function in a Rodent Model

**DOI:** 10.3390/nu13020704

**Published:** 2021-02-22

**Authors:** Miklós Sipos, Borbála Péterffy, Réka Eszter Sziva, Péter Magyar, Leila Hadjadj, Bálint Bányai, Anita Süli, Eszter Soltész-Katona, Dóra Gerszi, Judit Kiss, Mária Szekeres, György L. Nádasy, Eszter Mária Horváth, Szabolcs Várbíró

**Affiliations:** 1 Department of Obstetrics and Gynecology, Faculty of Medicine, Semmelweis University, Üllői Street 78/a, 1083 Budapest, Hungary; sipos.miklos.dr@gmail.com (M.S.); sziva.reka@semmelweis-univ.hu (R.E.S.); sulia1989@gmail.com (A.S.); gerszi.dora@med.semmelweis-univ.hu (D.G.); varbiro.szabolcs@med.semmelweis-univ.hu (S.V.); 2 Department of Physiology, Faculty of Medicine, Semmelweis University, Tűzoltó Street 37-47, 1094 Budapest, Hungary; peterffy.borbala@gmail.com (B.P.); banyai.balint@gmail.com (B.B.); soltesz-katona.eszter@med.semmelweis-univ.hu (E.S.-K.); szekeres.maria@med.semmelweis-univ.hu (M.S.); nadasy.gyorgy@med.semmelweis-univ.hu (G.L.N.); 3 Medical Imaging Centre, Faculty of Medicine, Semmelweis University, Üllői Street 78/a, 1083 Budapest, Hungary; drmagyarpeter@gmail.com; 4 Department of Translational Medicine, Faculty of Medicine, Semmelweis University, Tűzoltó Street 37-47, 1094 Budapest, Hungary; leila.hadjadj@gmail.com; 5 Department of Morphology and Physiology, Faculty of Health Sciences, Semmelweis University, Vas Street 17, 1088 Budapest, Hungary; kissjudit22@gmail.com

**Keywords:** Vitamin D, vascular function, gender difference, renal artery, rat model, endothelial dysfunction

## Abstract

Vitamin D deficiency shows positive correlation to cardiovascular risk, which might be influenced by gender specific features. Our goal was to examine the effect of Vitamin D supplementation and Vitamin D deficiency in male and female rats on an important hypertension target organ, the renal artery. Female and male Wistar rats were fed with Vitamin D reduced chow for eight weeks to induce hypovitaminosis. Another group of animals received normal chow with further supplementation to reach optimal serum vitamin levels. Isolated renal arteries of Vitamin D deficient female rats showed increased phenylephrine-induced contraction. In all experimental groups, both indomethacin and selective cyclooxygenase-2 inhibition (NS398) decreased the phenylephrine-induced contraction. Angiotensin II-induced contraction was pronounced in Vitamin D supplemented males. In both Vitamin D deficient groups, acetylcholine-induced relaxation was impaired. In the female Vitamin D supplemented group NS398, in males the indomethacin caused reduced acetylcholine-induced relaxation. Increased elastic fiber density was observed in Vitamin D deficient females. The intensity of eNOS immunostaining was decreased in Vitamin D deficient females. The density of AT_1_R staining was the highest in the male Vitamin D deficient group. Although Vitamin D deficiency induced renal vascular dysfunction in both sexes, female rats developed more extensive impairment that was accompanied by enzymatic and structural changes.

## 1. Introduction

The clear correlation between low plasma Vitamin D levels, hypertension and adverse cardiovascular events is widely described in the scientific literature, but randomized, controlled clinical studies failed to prove the direct advantageous effects of Vitamin D supplementation in cardiovascular prevention [1]. In the latest available study (VITAL), the combined administration of Vitamin D_3_ (2000 IU per day) and omega-3 fatty acids (1 g per day) had no effect on major and minor cardiovascular events during the 5-year-long follow-up, although it should be noted, that the placebo group was not Vitamin D deficient, as the average plasma 25-OH-D_3_ level was around 30 ng/mL [2] The discrepancy between these observations may be the results of the differential reactivity of patient subgroups.

Previous studies described differences in Vitamin D metabolism [3,4] and susceptibility to Vitamin D deficiency of the cardiovascular system in males and females [5]. Therefore, in our study we analyzed the possible gender differences in the effect of vitamin D deficiency and supplementation on renal arterial function. Renal arterial dysfunction plays an important role in the development of hypertension through its function in the regulation of renal blood flow, influencing renin-angiotensin-aldosterone axis [6]. In the current rodent study, the duration of diet-induced hypovitaminosis and Vitamin D supplementation was 8 weeks that allowed us to observe the early signs of the developing vascular changes [7,8,9]. Our goal was to model the appropriate Vitamin D supplementation and relevant Vitamin D deficiency in male and female rats, and to examine their effect on an important hypertension target organ, the renal artery. Our aim was to detect possible gender differences in Vitamin D dependent vascular changes.

## 2. Materials and Methods

### 2.1. Chemicals

Ex vivo functional measurements of isolated rat renal arteries were performed in freshly prepared Krebs-Ringer solution (in mmol/L): NaCl 119, KCl 4.7, NaH_2_PO_4_ 1.2, MgSO_4_ 1.17, NaHCO_3_ 24, CaCl_2_ 2.5, glucose 5.5 and EDTA 0.034. These solutions were kept at 37 °C, and bubbled (gas mixture containing O_2_ 20%, CO_2_ 5% and N_2_ 75%) for pH stabilization (Sigma-Aldrich, St Louis, MO, USA).

Phenylephrine, acetylcholine, indomethacin, the COX-2 inhibitor NS398, and DMSO were purchased from Sigma-Aldrich. 

### 2.2. Animals

The study was performed in accordance with the Guide for the Care and Use of Laboratory Animals published by the US National Institutes of Health (8th edition, 2011) and the EU conform Hungarian Law on Animal Care (XXVIII/1998). The institutional Animal Care Commission has confirmed the research protocol (IRB: 8/2014 PEI/001/1548-3/2014, PEI/001/820-2/2015).

The 21-28 days old female and male Wistar rats were delivered to the Animal Facility of Semmelweis University in agreement with Charles River Ltd. (Charles River Ltd., AnimaLab, Vác, Hungary). The female and male animals were randomly assigned to two further groups, resulting in four experimental groups; female Vitamin D supplemented group (FD+; *N* = 13), female Vitamin D deficient group (FD–; *N* = 11), male Vitamin D supplemented group (MD+; *N* = 13) and male Vitamin D deficient group (MD–; *N* = 11).

### 2.3. Chronic Treatment of the Rats

Vitamin D deficiency was induced by vitamin D intake reduction by feeding the rats with Vitamin D Free Lab Rat/Mouse Chow (Ssniff Spezialdiaten GmbH, Soest, Germany) containing less than 5 IU/kg Vitamin D_3_ ad libitum for eight weeks [7,9]. Vitamin D supplemented groups were fed by a regular chow containing 1000 IU/kg of Vitamin D ad libitum. Oral administration of additional Vitamin D through a gavage cannula to vitamin D supplemented animals ensured the target Vitamin D levels (25–50 ng/mL) of these rats (500 IU cholecalciferol on the second week and a weekly dose of 140 IU/100 g on the fifth, sixth and seventh weeks (Vigantol (cholecalciferol) 20,000 IU/mL, Merck/Merck Serono, Mumbai, India). The animals had access to tap water ad libitum. Rats were housed at constant room temperature (22 °C ± 1 °C) and had 12 h/12 h light-dark cycle. The average 25-OH-D_3_ levels in the experimental groups were the following: FD+: 32.328 ± 4.49 ng/mL; FD–: 6.044 ± 0.63 ng/mL; MD+: 19.66 ± 0.81 ng/mL; MD– rats: 3.59 ± 0.21 ng/mL [7,9]. All animals were normotensive [9,10]. Vitamin D status had no significant effect on final body weight, weight gain, serum progesterone or testosterone levels in any genders [7,9,10].

After the 8-week-long protocol, rats were anesthetized with Nembutal (45 mg/kg intraperitoneal injection). The cardiovascular system was perfused with heparinized nKR solution for 2 min. Renal arterial segment was cut into 5 (2 mm long) equal pieces. A total of 4 of these were placed on a conventional wire myograph setup (610-M MultiMyograph System; Danish Myo Technology, Hinnerup, Denmark/AnimaLab, Hungary). The 5th vascular ring, when available, was fixed in formalin and embedded in paraffin (*N* = 4–6 in each group).

### 2.4. Myography

Conventional wire myograph system was used to measure the isometric tension of isolated renal arterial rings. The organ chambers were filled with 8 mL nKR and kept at 37 °C. The 15 mN pre-tension was reached progressively. After the development of stable pre-tension, 124 mmol/L K^+^ was applied (3 min) to test the contractility of the vessels and to serve as the reference value for contraction force. Vascular rings were equilibrated in nKR and accumulative doses of phenylephrine (Phe), which is an α1-adrenergic receptor agonist (10^−9^–10^−6^ mol/L) or angiotensin II (10^−9^–10^−7^ mol/L) was administered to induce contraction. Acetylcholine (Ach) induced vasodilation was examined after Phe precontraction (10^−6^ mol/L) by incubating the vessels with increasing doses of Ach (10^−9^–10^−6^ mol/L). Phe-induced contraction and Ach-induced vasodilation was also examined after 30 min incubation with the cyclooxygenase-2 (COX-2) inhibitor NS398 (10^−5^ mol/L) or the COX inhibitor indomethacin (10^−4^ mol/L), or their vehicle dimethyl-sulfoxide (DMSO).

### 2.5. Immunohistochemistry

Paraffin-embedded tissue sections were stained with hematoxylin-eosin (HE) and resorcin-fuchsin (RF). Immunohistochemistry was performed against α-smooth muscle actin (α-SMA), endothelial nitric oxide synthase (eNOS) and angiotensin II receptor-1 (AT_1_R). After deparaffinization antigen retrieval was performed by heating the slides in citrate buffer (pH = 6). Endogenous peroxidase activity was blocked by 3% H_2_O_2_ in dH_2_O. 2.5% normal horse serum (Vector Biolabs, Burlingame, CA, USA) was used to avoid non-specific labeling. Primary antibodies (α-SMA: 1:10,000; eNOS: 1:1000, (Abcam, Cambridge, UK), AT_1_R: 1:500 (Sigma-Aldrich, St. Louis, MI, USA)) were applied overnight at 4 °C. Horseradish peroxidase-linked anti-mouse or anti-rabbit polyclonal horse antibody (Vector Biolabs, Burlingame, CA, USA) was used for secondary labeling. Brown colored diamino-benzidine (DAB) was used for the visualization of specific labeling (Vector Biolabs, Burlingame, CA, USA). Blue colored hematoxylin served as counterstaining (Vector Biolabs, Birmingham, CA, USA). Light microscopy images were taken with Nikon ECLIPSE NI-U microscope and Nikon DS-Ri2 camera (Nikon, Minato City, Tokyo, Japan). The intima-media ratio of the vessels was calculated based on the measurement of intimal and media areas using the resorcin-fuchsin stained sections by ImageJ software (National Institutes of Health (NIH), Bethesda, MA, USA). The number of smooth muscle cell nuclei in the media layer of arteries was measured and their density per 1000 µm^2^ was calculated. In order to assess the density of elastic fibers, the non-calibrated optical density of the media layer of resorcin-fuchsin stained vessels was assessed. In case of immunohistochemical labeling, non-calibrated optical density of specific staining was measured in the intimal or media layers of the vessel walls using the ImageJ software.

### 2.6. Statistics

Vascular function curves were analyzed by repeated measures two-way ANOVA using Bonferroni’s post hoc test by Prism 8 (GraphPad Software, San Diego, CA, USA). Histological evaluations of the experimental groups were compared using Kruskal-Wallis test with Dunn’s multiple comparison test. *p* < 0.05 was uniformly accepted as the threshold for statistical significance. Each experimental group is presented in a different color; Vitamin D supplemented female group in red, Vitamin D deficient female group in orange, Vitamin D supplemented male group in blue and Vitamin D deficient male group in green. Gender difference is highlighted by violet, while burgundy color indicates significant difference due to different Vitamin D status. Raw study data are available as supplementary material, Table S1.

## 3. Results

### 3.1. Vascular Function of Renal Arteries

**Figure 1:**
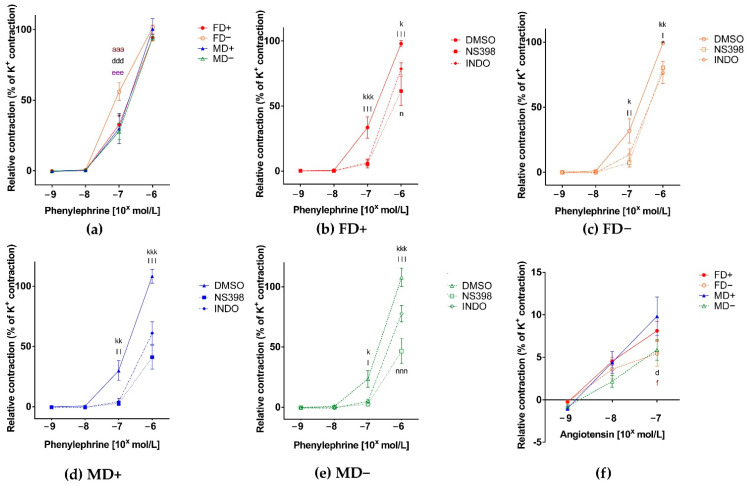
**Contraction ability of isolated renal artery segments:** (**a**) Phenylephrine (Phe) induced contraction. Vitamin D deficient female rats showed significantly increased contraction at Phe concentration of 10^−7^ mol/L compared to their Vitamin D supplemented and male counterparts. Data are shown as mean ± SEM; *N* = 8 in each group; **aaa**: *p* < 0.001 FD+ vs. FD–, ddd: *p* < 0.001 FD– vs. MD+, **eee**: *p* < 0.001 FD– vs. MD–; **Phe-induced contraction in the presence of COX-2 inhibitor (NS398) or general COX inhibitor (indomethacin; INDO), or their vehicle DMSO** (**b**) **in female Vitamin D supplemented rats:** Both general COX, and specific COX-2 inhibition led to decreased contraction that was more pronounced in case of specific COX-2 inhibition; (**c**) **in female Vitamin D deficient rats:** Both indomethacin and NS398 pretreatment resulted in reduced contraction force; (**d**) **in male Vitamin D supplemented rats.** Similarly to female Vitamin D supplemented group, both general COX, and specific COX-2 inhibition led to reduced contraction with a more pronounced effect of NS398; (**e**) **in male Vitamin D deficient rats**. Also in this experimental group, both indomethacin and NS398 caused reduced contraction with a significantly bigger inhibition by COX-2 blocker. Data are shown as mean ± SEM; *N* = 8 in each group; kkk: *p* < 0.001, kk: *p* < 0.01, k: *p* < 0.05 INDO vs. DMSO; lll: *p* < 0.001, ll: *p* < 0.01, l: *p* < 0.5 NS398 vs. DMSO; nnn: *p* < 0.001, n: *p* < 0.05 NS398 vs. INDO; (**f**) **Angiotensin II-induced contraction of isolated renal artery segments.** Both Vitamin D deficient experimental groups showed decreased contraction at angiotensin concentration of 10^−7^ mol/L compared to Vitamin D supplemented males. Data are shown as mean ± SEM; *N* = 7–9 in each group; d: *p* < 0.05 FD– vs. MD+, **f**: *p* < 0.05 MD+ vs. MD–.

The contraction ability of isolated renal artery segments in increasing concentrations of phenylephrine was maintained in all experimental groups. Vitamin D deficiency in female rats, but not in males, resulted in increased reactivity to phenylephrine, showed by the increased Phe-induced contraction of FD– rats’ vessels (Figure 1. Panel a). Both general COX and specific COX-2 inhibition decreased the Phe evoked contraction in all experimental groups. Specific COX-2 inhibition resulted in significantly greater inhibition compared to indomethacin in a FD+ and MD– experimental groups (Figure 1, Panel b–e). Vitamin D deficiency resulted in reduced angiotensin II-induced contraction in both sexes (FD– and MD–) compared to Vitamin D supplemented males (MD+) (Figure 1, Panel f).

**Figure 2:**
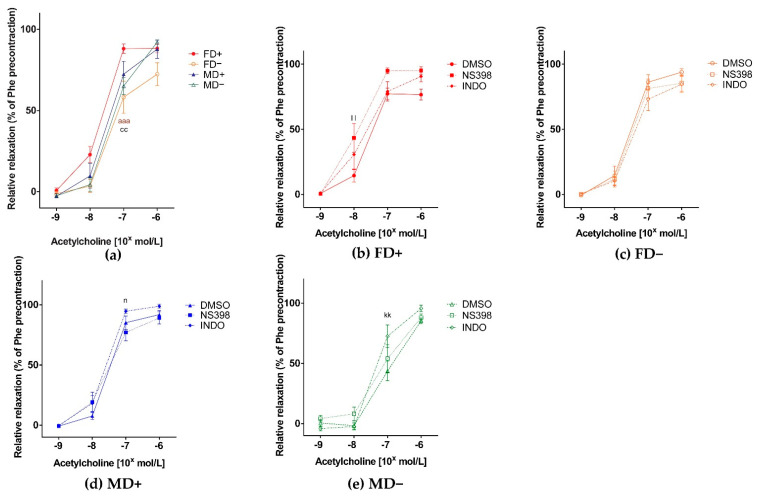
**Relaxation ability of renal arteries:** (**a**) **Acetylcholine-induced relaxation of isolated renal artery segments.** Significantly reduced relaxation was observed in FD– and MD– group compared to the FD+ group at 10^−7^ mol/L Ach concentration. Data are shown as mean ± SEM; *N* = 8 in each group; **aaa**: *p* < 0.001 FD+ vs. FD–; cc: *p* < 0.01 FD+ vs. MD–; **Ach-induced relaxation in the presence of COX-2 inhibitor (NS398) or general COX inhibitor (indomethacin; INDO), or their vehicle DMSO** (**b**) **in female Vitamin D supplemented rats.** Specific COX-2 inhibition resulted in increased relaxation in FD+ experimental group; (**c**) **in female Vitamin D deficient rats.** There was no significant difference in relaxation in the presence of general COX or specific COX-2 inhibitor; (**d**) **in male Vitamin D supplemented rat.** General COX inhibition led to enhanced relaxation compared to specific COX-2 inhibition in MD+ experimental group; (**e**) **in male Vitamin D deficient rats.** In this experimental group, more pronounced relaxation occurred in the presence of indomethacin. Data are shown as mean ± SEM; *N* = 7–8 in each group; kk: *p* < 0.01, INDO vs. DMSO; ll: *p* < 0.01 NS398 vs. DMSO; n: *p* < 0.05 NS398 vs. INDO.

In concordance to the increased Phe-induced contraction of Vitamin D deficient female renal arteries, we observed decreased Ach-induced relaxation of these vessels, suggesting the presence of reduced endothelial dependent relaxation mechanisms in these vessels (Figure 2, Panel a). Specific COX-2 inhibition led to more pronounced Ach dependent relaxation in the FD+ experimental group. In males (MD+, MD–), we found a more pronounced Ach dependent relaxation in the presence of indomethacin. In the MD+ group, the difference was significant compared to specific COX-2 inhibition, in the MD– group compared to the DMSO treated control (Figure 2, Panel b–e).

### 3.2. Histology of Renal Arteries

**Figure 3:**
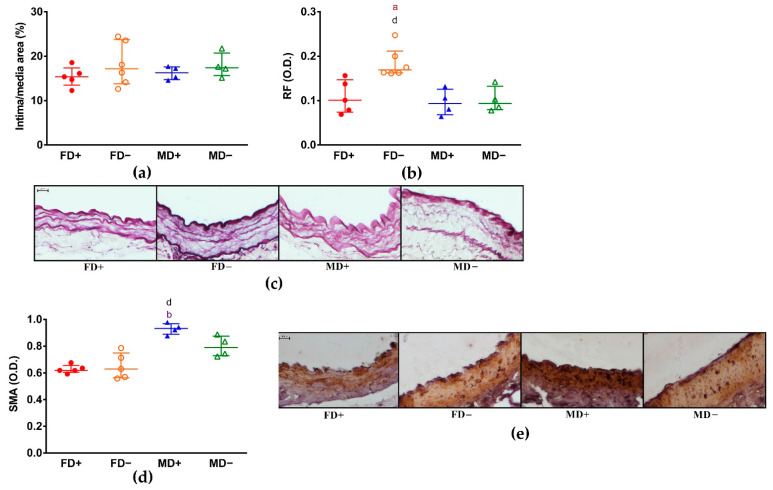

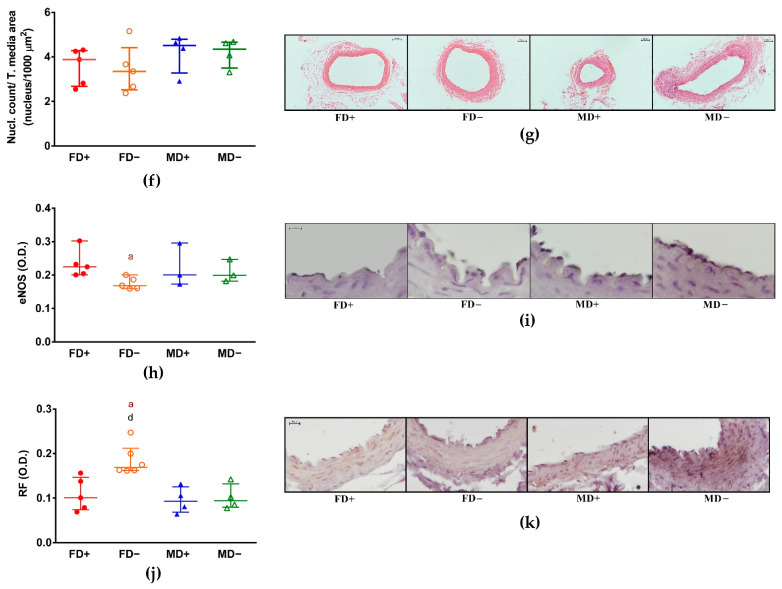
**Histology of the renal arteries:** (**a**) **Intima/media ratio of the renal arteries measured on resorcin-fuchsin stained tissue sections.** There were no significant effect of gender or Vitamin D deficiency on intima/media ratio, neither was any intergroup difference; (**b**) **Density of resorcin-fuchsin staining in the media layer of renal arteries.** FD– group had significantly augmented staining intensity compared to the FD+ and MD+ groups; (**c**) **Representative images of resorcin-fuchsin stained renal arteries** (scale bar is 20 µm); (**d**) **α-smooth muscle actin (α-SMA) immunohistochemistry of renal arteries.** The MD+ group showed significantly higher SMA staining intensity compared to the FD+ and FD– groups; (**e**) **Representative images of renal arterial sections stained against α-SMA** (scale bar is 20 µm). In all immunohistochemical stainings, brown-colored diamino-benzidine represents specific labeling and blue-colored hematoxylin was used for counterstaining; (**f**) **Density of smooth muscle cell nuclei in the media layer of renal arteries.** Between experimental groups, there were no significant difference in the number of smooth muscle nuclei per 1000 µm^2^. (**g**) **Representative images of hematoxylin-eosin stained tissue sections** (scale bar is 100 µm). (**h**) **eNOS immunohistochemistry of renal arteries.** eNOS specific labeling intensity was significantly lower in FD– rats compared to the FD+ animals; (**i**) **Representative images of renal arterial sections stained against eNOS** (scale bar is 10 µm); (**j**) **Angiotensin II receptor-1 (AT_1_R) immunohistochemistry of renal arteries.** AT_1_R specific staining showed the highest intensity in MD– experimental group, it differed significantly from the staining intensity measured in FD– animals; (**k**) **Representative images of renal arterial sections stained against AT_1_R** (scale bar is 20 µm). Data are shown as median [IQR]; *N* = 3–6 in each group; a: *p* < 0.05, aa: *p* < 0.01 FD+ vs. FD–; b: *p* < 0.05 FD+ vs. MD+; d: *p* < 0.05 FD– vs. MD+; e: *p* < 0.05 FD– vs. MD–.

We did not observe structural changes of these vessels by measuring intima/media ratio (Figure 3, Panel a,b). However, the increased density of elastic fibers in the media layer of FD– renal arteries showed by the increased intensity of resorcin-fuchsin staining may have contributed to the increased contractile force and the reduced relaxation ability of these vessels (Figure 3, Panel c). On the other hand, the degree of α-SMA staining intensity was significantly higher in Vitamin D supplemented male animals compared to both female groups that was not associated to any measured vascular function (Figure 3, Panel d,e). The density of smooth muscle cell nuclei in the media layer was similar in all experimental groups (Figure 3, Panel f,g). The increased contraction force and reduced relaxation ability of FD– renal arteries was accompanied by reduced staining intensity of eNOS specific labeling of the endothelial layer confirming the role of developing endothelial dysfunction in these animals (Figure 3, Panel h,i). AT_1_R specific staining showed significantly higher intensity in Vitamin D deficient males (MD–) compared to Vitamin D deficient females (FD–) (Figure 3, Panel j,k).

## 4. Discussion

In our study, the body weight-adjusted Vitamin D supplementation resulted in lower 25-OH-D_3_ level in the male group compared to females. Several previous studies reported lower Vitamin D levels in men compared to women and also the prevalence of Vitamin D deficiency was found to be higher in males [3,4,11,12]. In these clinical studies, the Vitamin D intake was not normalized, so the measured difference could be the result of body weight variations or lifestyle differences. Still, in our animal model, where the Vitamin D supplementation was adjusted to body weight, the observed gender difference was still present. It may support the proposition that the bioavailability, the absorption, and the metabolism of orally administered Vitamin D_3_ have gender-related differences. Testosterone was also found to increase the expression of Vitamin D binding protein in male rats [13]. In a recent study, DHT was shown to induce CYP27B1 and block CYP24A1. CYP27B1 codes a hydroxylase that converts 25-OH-Vitamin D_3_ to its active form, calcitriol. CYP24A1 codes another hydroxylase that converts calcitriol into an excretable form. These mechanisms both point to having lower detectable serum Vitamin D levels even though we cannot see its deteriorating effect [14].

In our model, Vitamin D deficient females showed increased phenylephrine-induced contraction and decreased acetylcholine-induced relaxation, which was accompanied by reduced intensity of eNOS immunostaining. The positive effect of Vitamin D on vascular relaxation is supported by observations describing that both in vitro and in vivo Vitamin D treatment can enhance acetylcholine-induced relaxation, and the positive effect of Vitamin D on vessel function can be traced back partially to enhanced eNOS expression and NO production [15,16]. In the lack of Vitamin D, its eNOS-expression increasing effect is also missing [17,18]. This reduced eNOS expression leads to the deterioration of NO-dependent relaxation mechanisms.

When the constrictor/relaxant balance moves towards the constrictors, it can lead to increased phenylephrine-induced contraction as well as decreased acetylcholine-induced relaxation as seen in Vitamin D deficient female rats. In contrast, in Vitamin D deficient males, only the impairment of acetylcholine-induced relaxation was observed, the increase in phenylephrine-induced contraction was not present. Neither did we observe the decrease in eNOS immunostaining intensity. Although the expression of the enzyme might not change, the reduced bioavailability of NO could play role in the decreased relaxing capacity. An increase in the production of oxygen-derived free radicals may reduce the bioavailability of NO. The produced superoxide forms peroxynitrate with NO in a spontaneous reaction reducing its concentration. Peroxynitrate itself is a potent oxidant that can further deteriorate vascular function [19,20]. Besides, Vitamin D itself has antioxidant properties [21], it can also reduce the level of peroxynitrate [22]. Therefore, the decreased relaxation in the Vitamin D deficient male group can be the consequence of the decreased bioavailability of NO.

The angiotensin II-induced contraction was the most intensive in the Vitamin D supplemented male group, while in the Vitamin D deficient groups the contraction was significantly lower. Based on α-SMA immunohistochemistry, the amount of smooth muscle actin is bigger in the MD+ group, than in the female groups. According to the literature, androgens enhance the proliferation of the smooth muscle cells [23], while estrogens inhibit it [24]. Moreover, estrogens decrease, but testosterone increases the vascular response to angiotensin II [25]. The possible gender difference in the amount of smooth muscle elements can explain the intensive angiotensin II-induced vascular vasoconstriction in the MD+ group. On the other hand, in the MD– group neither the angiotensin II-induced contraction, nor the intensity of α-SMA immunohistochemical labeling was ignificantly different from the female groups. It may suggest that in case of Vitamin D deficiency there is no gender difference in the amount of smooth muscle elements. It may also contribute to the observation that the angiotensin II-induced vascular response was less intense in the MD– group than in Vitamin D supplemented male group. However, the AT_1_R density was the highest in this (MD–) group. In the regulation of the renin-angiotensin-aldosterone system (RAAS) estrogen, testosterone, as well as Vitamin D play important roles. While estrogen decreases the synthesis of angiotensin converting enzyme, testosterone increases it [25]. In the lack of Vitamin D, the activity of RAAS is increased [26]. The expression of AT_1_ receptor is decreased by both estrogen and Vitamin D [15,27]. This can explain our result that the most intensive AT_1_R specific staining was found in Vitamin D deficient males. The lowest optical density was measured in Vitamin D deficient females. As the amount of smooth muscle elements is lower in females, the amount of receptors by tissue unit might be also lower, which may explain the lower receptor density.

Both the selective COX-2 and the non-selective COX inhibition of prostanoid pathway decreased the phenylephrine-induced vasoconstriction in all experimental groups. However, the selective COX-2 and the non-selective COX-inhibitor had diverse effect on acetylcholine-induced relaxation in different genders. The selective COX-2 inhibitor increased the acetylcholine-induced relaxation in Vitamin D supplemented females. In the male groups, a non-selective COX inhibition caused increased relaxation. In male Vitamin D supplemented rats, general COX inhibition led to enhanced relaxation compared to specific COX-2 inhibition, while in male Vitamin D deficient rats more pronounced relaxation occurred compared to control (DMSO). In the female groups, the effect of COX-2 inhibition is more pronounced, while in males, the general inhibition of COX isoforms by indomethacin led to the same result. This can be caused by the estrogen mediated enhancement of COX-2 expression [28,29], leading to the increased impact of COX-2-related processes in females. The decreased contraction and enhanced vasodilation in the presence of COX-inhibitors, suggest a COX-inhibition caused absence of a vasoconstrictor mediator. The cyclooxygenase enzyme produces endoperoxides, e.g., prostaglandins (PGH_2_) from arachidonic acid, which causes smooth muscle contraction itself. The endoperoxides can transform further to prostacyclin (PGI_2_), thromboxane A_2_ (TXA_2_), prostaglandin D_2_, prostaglandin E_2_, prostaglandin F_2α_, etc. with the help of their own synthases, among which prostacyclin synthase is the dominant in the endothelium [30]. The PGI_2_ acting on its own receptor causes vasorelaxation [31]. On the other hand, in various pathological conditions like hypertension or diabetes, besides PGH_2_, TXA_2_ and other prostanoids, prostacyclin can also lead to vasoconstriction, when binding to the thromboxane-prostanoid receptor (TP). In the production of these endothelium-derived contracting factors (EDCF) both COX-1 and COX-2 can play a role [30,32,33]. The importance of the cyclooxygenase-derived products in renal arterial function was highlighted in a study, which observed that Vitamin D treatment restored the renal artery function in estrogen-deficient rats by decreasing COX-2 and TP receptor expression [3].

In our study, resorcin-fuchsin staining was used to label the elastic fibers of the examined renal arterial tissue sections. Although we found no changes in the ratio of the tunica intima and media thickness, increased elastic fiber density was detected in the female Vitamin D deficient group. This may represent the first sign of increased vascular wall rigidity. It was previously described that Vitamin D inhibits the synthesis of elastin [34], furthermore the calcification of the coronary arteries are more severe in case of low serum 25-OH-D_3_ level [35]. While we could not find any morphological changes in the renal artery of Vitamin D deficient males, we detected thickening of tunica media, decrease in the ratio of the intima/media and narrowing of the vascular lumen in cerebral arteries before [7]. When hyperandrogenic status accompanied the Vitamin D deficiency these changes appeared also in females [8], suggesting a possible gender specific regional difference in vascular remodeling induced by Vitamin D deficiency.

According to our results, 8-week-long Vitamin D deficient state of young, healthy rats leads to early impairment of vascular function in both sexes that could contribute to increased cardiovascular risk later in life. In contrast, a recent meta-analysis summarizing human interventions failed to show the beneficial effect of Vitamin D supplementation on cardiovascular risk [36]. However, several differences should be considered before comparing these results. First, the average age of the subjects was 66 and most studies only involved patients with preexisting cardiovascular illness. These circumstances suggest that Vitamin D was a proposed treatment to preexisting conditions in human studies while our Vitamin D supplementation rather serves a preventive measure. Furthermore, in human studies it is difficult to separate Vitamin D deficient and supplemented subgroups. The unknown duration of previous hypovitaminosis can also be a limitation of these studies.

According to the literature, there are several gender specific consequences of Vitamin D deficiency: while in males, the prevalence is higher, females seem to be more sensitive to Vitamin D deficiency. A Swiss cohort study found inverse correlation between cardiovascular mortality and 25-OH-D_3_ levels in women, while this relation was not present in men [5]. According to the prospective MONICA (Monitoring of Trends and Determinants of Cardiovascular Disease) study, an increased cardiovascular mortality can be found in Vitamin D deficient women compared to men [37].

The interactions between sex steroids and Vitamin D are assumed to cause the observed differences. In vitro Vitamin D can modify the effects of both estrogens and testosterone in vascular smooth muscle cells and endothelial cells [38]. Vitamin D and estrogens are mutually affect the expression of each other’s receptors. Vitamin D analogues enhance the expression of estrogen-receptor-α (ERα) [39,40], while estrogen analogues enhance the expression of Vitamin D (VDR) receptor in vascular smooth muscle cells [38,40]. Therefore, they mutually enhance the sensitivity of vessels to Vitamin D and estrogen. It is possible, that they can even enhance each other’s vasoprotective effects this way [39]. Furthermore, Vitamin D also increases androgen receptor (AR) expression, but testosterone seems to have no effect on VDR expression [41,42]. Based on the above detailed interactions, one can hypothesize that in the lack of Vitamin D, the protective effects of estrogen are compromised that can contribute to the increased sensitivity to Vitamin D deficiency of females. Our results also reflect the enhanced sensitivity of female rats to early vascular changes caused by Vitamin D deficiency. Although relaxing dysfunction emerged in both sexes, the contraction hyperreactivity was only present in females, where also enzymatic and morphological differences were detected.

## 5. Conclusions

In our present study, the 8-week-long Vitamin D supplementation resulted in lower 25-OH-D_3_ level in male rats compared to females, despite the weight-adjusted dosage. This may raise the possibility that males need higher dose of Vitamin D supplementation in order to reach the same serum Vitamin D level; however, further investigations are needed to prove this hypothesis. Our results confirm that Vitamin D deficiency increases cardiovascular risk in both sexes. We found vascular relaxing dysfunction in both sexes. In case of females, this is accompanied by enhanced phenylephrine-induced contraction, together with enzymatic and morphological changes. By contrast, in males presumably the decreased bioavailability of NO is the main cause of the impaired relaxation. The vascular dysfunction of the renal artery can cause decreased renal perfusion in both sexes in the long term, affecting the function of the renin-angiotensin-aldosterone system that can contribute to further vascular dysfunction and hypertension. As female rats showed more advanced changes, women are supposed to be more sensitive to the early effects Vitamin D deficiency, so it is extremely important to maintain the optimal Vitamin D level. However, Vitamin D supplementation is not negligible in males either, as they are predestined to higher cardiovascular risk, so prevention has high importance for them.

## Data Availability

Study data can be found in Table S1.

## Supplementary Materials

The following are available online at https://www.mdpi.com/2072-6643/13/2/704/s1, Table S1: Study data.
