# Supplementary material for: Vitamin D Deficiency Cause Gender Specific Alterations of Renal Arterial Function in a Rodent Model
Source: Nutrients. 2021 Feb 22;13(2):704. doi: 10.3390/nu13020704 (PMC7926839; doi:10.3390/nu13020704)
Supplement: Supplementary file 1 [file nutrients-13-00704-s001.pdf]

Phenylephrine induced contraction

Phenylephrin doses (10^M/L)	Female D+								
-9.000000	-0.545310	0.000584	-0.066870	-0.230170	-0.261810	0.095587	0.019915	-0.100200	
-8.000000	1.169241	2.043211	-0.020110	-0.280620	0.312262	0.240694	0.066161	0.000991	
-7.000000	52.399930	50.944220	8.483752	27.033090	36.298040	24.805900	12.116860	49.463170	
-6.000000	104.912600	103.703700	90.218410	86.295300	86.678030	96.157010	89.514000	97.719790	
	Female D-								
-9.000000	0.059914	-0.253820	-0.134540	-0.279680	0.209893	-0.072960	-0.143600	-0.418950	
-8.000000	0.699392	1.943906	-0.140790	0.891275	0.112556	0.076084	0.130640	0.711996	
-7.000000	73.697370	79.922420	30.371270	49.094990	50.143200	45.289510	44.807810	74.580440	
-6.000000	105.594800	108.324600	100.175400	95.222530	98.779700	95.781140	100.919000	106.781000	
	Male D+								
-9.000000	-0.723860	-0.378580	0.159614	-0.345980	-0.309630	-0.653530	-0.543330	-0.820010	
-8.000000	-1.110800	1.143334	1.285507	-0.186320	-0.020110	1.777075	-0.284910	-1.053130	
-7.000000	8.786680	64.516460	28.299000	13.685430	10.392810	88.534970	18.223190	7.034903	
-6.000000	108.279400	123.589900	85.919980	73.134080	112.908700	127.475400	79.842890	91.977140	
	Male D-								
-9.000000	0.460689	-0.520790	-0.060190	-0.359720	0.072120	-0.931320	-0.262100	-0.036630	
-8.000000	2.929500	-0.506650	1.101307	0.903816	0.189425	-0.649110	-0.038960	0.919292	
-7.000000	60.297220	20.262730	22.930220	20.017400	16.942010	21.763440	15.338970	45.359970	
-6.000000	101.279300	97.624240	90.050130	87.341280	101.161900	96.439710	86.759440	94.556900	

Phenylephrine induced contraction in the presence of DMSO. NS398 and indomethacin in Female D+

Phenylephrin doses (10^M/L)	DMSO							
-9.000000	0.284070	-0.195259	0.095124	0.318250	-0.355400	-0.875630	-0.615540	3.170867
-8.000000	0.119253	1.156443	0.429817	0.017930	0.474767	-0.250310	-0.491150	3.613957
-7.000000	2.545943	25.041940	51.788980	27.740430	51.468730	67.765960	4.882590	36.580360
-6.000000	100.040500	83.084550	99.611950	102.321900	98.257990	105.365300	96.130200	98.518420
	NS398							
-9.000000	0.131846	-0.123665	-0.076536	0.175248	-0.262340	-0.376310	-0.382260	2.697023
-8.000000	1.289157	0.135284	-0.086926	-0.456632	-0.417120	-0.570940	-0.456130	2.120239
-7.000000	25.878360	5.891922	0.711335	2.613911	0.451780	0.910825	0.988274	5.985138
-6.000000	94.911740	59.314280	94.093220	37.660230	24.197730	81.001620	17.056690	83.691770
	INDO							
-9.000000	-0.118703	-0.185216	-0.019380	-0.419680	-0.831820	-0.133540	-0.052790	3.707484
-8.000000	1.545102	-0.196883	-0.181800	-0.715704	-1.253860	-0.032270	-0.164410	3.406460
-7.000000	23.756320	1.291649	9.036462	5.819313	-0.731600	2.131093	1.498571	9.189380
-6.000000	71.062930	93.202920	83.478370	50.153260	83.312360	85.160250	75.960270	86.011390

Phenylephrine induced contraction in the presence of DMSO. NS398 and indomethacin in Female D-

Phenylephrin doses (10^M/L)	DMSO							
-9.000000	-0.199570	-0.313620	0.139095	0.265880	-0.278830	-0.011950	0.462620	-0.343330
-8.000000	-0.199570	-0.295700	2.074060	4.548175	0.311035	0.000664	1.373227	0.039940
-7.000000	10.011010	19.245850	70.743690	7.679747	47.547460	6.849088	25.454690	66.522010
-6.000000	91.587140	98.939840	98.445230	100.281300	101.717000	97.626230	100.507300	105.236700
	NS398							
-9.000000	-0.002220	0.033791	-0.068200	-0.479080	0.467322	-0.260740	-0.254070	-0.483310
-8.000000	-0.458800	0.222862	-0.777770	0.729452	-0.303250	-0.369430	-0.076370	0.059050
-7.000000	7.985726	6.570817	11.305810	0.146488	1.768983	0.440392	2.034541	28.647440
-6.000000	90.196820	84.179190	80.781440	50.900230	80.829670	69.818500	96.128080	89.509700
	INDO							
-9.000000	-0.035560	-0.320700	-0.233460	-0.228100	0.083782	-0.530350	-0.701110	-0.480540
-8.000000	0.305078	-0.291380	-0.482150	0.008507	-0.213420	-1.779840	-0.341790	-0.490960
-7.000000	24.549400	21.160560	2.109412	-0.039880	0.612934	9.352527	18.589660	32.363920
-6.000000	91.863030	99.314620	75.313290	73.630480	29.069840	56.708850	102.456300	85.618240

Phenylephrine induced contraction in the presence of DMSO. NS398 and indomethacin in Male D+

Phenylephrin doses (10^M/L)	DMSO							
-9.000000	0.316525	-0.124860	0.205306	-0.135610	-0.313150	-0.058130	0.144626	-0.447380
-8.000000	0.841869	0.210378	2.536034	-0.104670	0.256619	0.085771	0.255661	0.026316
-7.000000	16.123740	49.741570	59.093620	1.781172	55.661490	2.540253	31.016210	23.398660
-6.000000	124.056700	102.659200	125.807100	85.267330	117.057500	88.023030	122.139000	101.433400
	NS398							
-9.000000	-1.109640	-0.465270	-0.426720	-0.245510	-0.835980	-0.114060	-0.064440	-0.064200
-8.000000	-0.978700	-0.635090	-0.384670	-0.430010	-0.834220	-0.353100	-0.172560	-0.133150
-7.000000	-0.045900	4.399107	0.191485	-0.000980	16.078090	1.246016	0.201199	0.592033
-6.000000	59.850430	35.202160	23.407180	64.921070	90.180340	22.104870	19.055870	13.102580
	INDO							
-9.000000	-0.499120	-1.294320	-0.726640	-0.212650	-0.350450	-0.077080	-0.226670	-0.356380
-8.000000	-0.709690	-0.567590	-1.179910	-0.356350	-0.469510	-0.266100	-0.115100	-0.461710
-7.000000	2.312886	22.777830	0.940851	1.966175	0.298654	2.754583	0.099564	1.740893
-6.000000	77.972990	101.552500	32.940430	31.951390	46.406400	90.012120	60.539620	46.483010

Phenylephrine induced contraction in the presence of DMSO. NS398 and indomethacin in Male D-

Phenylephrin doses (10^M/L)	DMSO							
-9.000000	-0.003894	-0.092975	-0.974558	-0.002965	-0.108840	0.032634	-0.088260	0.011245
-8.000000	0.246317	0.010176	6.150177	0.077692	0.006803	0.165129	0.882620	-0.216809
-7.000000	41.096070	6.700249	21.547250	33.198310	15.369610	12.186220	57.546840	0.783569
-6.000000	128.257400	77.793660	95.455180	123.462500	116.818600	85.145610	138.041800	97.582720
	NS398							
-9.000000	0.338250	-0.202905	-0.618747	-0.289499	-0.018580	-0.112610	-0.841890	-0.087160
-8.000000	1.426403	-0.161402	-0.649063	-0.246485	0.291749	-0.140620	-0.374170	-0.130281
-7.000000	13.950580	0.320169	0.051190	-0.030448	0.205426	0.725947	5.425530	0.906001
-6.000000	60.360270	14.182290	37.255550	27.830110	80.122820	6.662704	58.464760	87.015060
	INDO							
-9.000000	1.004111	-2.109321	-0.760446	-0.407289	-0.375340	-0.961120	-0.029630	-0.265799
-8.000000	1.134828	-1.741519	-0.937395	0.006266	-0.330540	-2.165940	-0.301920	-0.175039
-7.000000	11.646230	6.587198	5.808222	11.762310	0.734454	1.002118	1.641507	1.044129
-6.000000	100.327200	85.613630	71.465560	98.975510	56.210810	75.910450	46.027790	86.644070

Angiotensin induced contraction

Angiotensin doses [10^M/L]	Female D+								
-9.000000	-0.399861	-0.482124	-0.133725	-0.803881	-0.161980	0.196952	-0.365090	-0.024797	
-8.000000	4.997482	5.681393	5.510007	2.998347	3.588939	3.441355	6.396008	3.560185	
-7.000000	11.358780	8.550951	10.034880	5.877763	5.664143	4.106244	13.083180	6.190019	
	Female D-								
-9.000000	-0.107575	-0.141440	-0.577647	-0.076892	0.084208	-0.206470	-0.705490	-0.033100	
-8.000000	4.442667	5.393408	0.609654	10.149860	0.802989	3.682717	1.076704	2.580144	
-7.000000	2.679058	9.556670	3.246236	13.394890	4.191178	5.857550	1.179393	3.244257	
	Male D+								
-9.000000	-0.802310	-1.284019	-0.640504	-0.621853	-2.665910	-0.379440	-0.740120	-0.820514	
-8.000000	7.648244	0.038248	3.632978	1.092423	11.017020	4.469401	3.234846	4.038786	
-7.000000	20.987350	5.481406	6.891623	3.307352	17.247840	6.826884	5.188382	12.664650	
	Male D-								
-9.000000	-0.016602	-0.520544	-0.186691	-0.402078	-0.425400	-1.145820	-2.123300	-1.466745	
-8.000000	1.735923	1.412232	3.840029	0.021665	0.329330	0.699419	4.784204	4.691291	
-7.000000	2.825187	2.951923	10.689020	8.489684	3.435094	2.577862	10.344270	5.614244	

Acetylcholine induced relaxation

Acetylcholine doses [10^M/L]	Female D+								
-9.000000	-3.760000	0.100000	0.780000	1.250000	-3.110000	-0.780000	13.690000	-1.740000	0.270000
-8.000000	15.080000	6.680000	17.410000	35.650000	7.780000	41.820000	47.100000	22.190000	12.080000
-7.000000	88.480000	87.230000	96.140000	93.880000	68.230000	90.500000	92.090000	94.080000	82.040000
-6.000000	85.830000	91.730000	97.250000	97.380000	76.280000	87.910000	82.450000	95.230000	80.040000
	Female D-								
-9.000000	-0.060000	-0.950000	0.210000	-4.070000	-5.720000	5.190000	-3.020000		
-8.000000	1.860000	-0.430000	1.790000	0.610000	-2.670000	26.990000	-3.380000		
-7.000000	47.230000	27.300000	33.750000	93.760000	65.810000	90.110000	49.390000		
-6.000000	57.800000	57.580000	48.530000	98.190000	84.140000	87.410000	73.030000		
	Male D+								
-9.000000	-1.650000	-0.050000	-3.280000	-3.240000	-1.230000	-2.690000	-7.110000		
-8.000000	2.280000	1.720000	-0.170000	55.260000	5.990000	3.590000	-0.170000		
-7.000000	63.100000	41.880000	58.700000	99.030000	71.870000	98.320000	73.710000		
-6.000000	77.190000	59.970000	85.960000	99.860000	94.450000	99.190000	98.060000		
	Male D-								
-9.000000	0.520000	-2.280000	-4.950000	-3.290000	-0.970000	-8.450000	-0.210000	-4.820000	2.230000
-8.000000	33.430000	-0.950000	-5.720000	6.500000	2.630000	-5.980000	8.670000	-4.980000	5.870000
-7.000000	85.910000	64.140000	81.550000	71.170000	40.910000	43.670000	76.910000	64.060000	57.440000
-6.000000	96.650000	91.770000	90.230000	90.130000	93.440000	92.950000	89.920000	97.590000	86.310000

Acetylcholine induced relaxation in the presence of DMSO. NS398 and indomethacin in Female D+

Acetylcholine doses [10^M/L]	DMSO						
-9.000000	1.400000	-0.190000	1.260000	3.000000	-2.390000	2.060000	0.520000
-8.000000	4.190000	37.040000	17.730000	26.280000	0.710000	6.320000	8.730000
-7.000000	76.090000	90.510000	85.240000	78.560000	55.470000	82.760000	72.360000
-6.000000	80.480000	91.950000	75.040000	77.220000	62.110000	86.870000	62.750000
	NS398						
-9.000000	-0.850000	2.080000	6.450000	-2.060000	-3.090000	0.560000	0.040000
-8.000000	12.850000	54.390000	57.990000	72.420000	7.820000	77.630000	20.890000
-7.000000	97.830000	97.940000	88.970000	99.430000	89.720000	102.580000	87.210000
-6.000000	98.500000	100.780000	94.000000	99.990000	79.090000	101.360000	92.030000
	INDO						
-9.000000	0.770000	-2.780000	3.140000	0.070000	-1.690000	6.140000	1.600000
-8.000000	52.260000	-3.160000	16.980000	41.320000	14.410000	84.390000	7.960000
-7.000000	97.950000	56.300000	67.430000	95.540000	82.850000	98.960000	54.520000
-6.000000	98.840000	82.750000	93.560000	100.920000	88.820000	99.480000	69.960000

Acetylcholine induced relaxation in the presence of DMSO. NS398 and indomethacin in Female D-

Acetylcholine doses [10^M/L]	DMSO						
-9.000000	-1.470000	-4.160000	0.760000	-0.640000	0.910000	-0.730000	1.100000
-8.000000	-1.910000	42.230000	5.660000	-3.060000	10.710000	40.050000	7.850000
-7.000000	61.680000	97.440000	96.420000	86.760000	68.940000	94.470000	97.540000
-6.000000	89.120000	97.220000	97.980000	95.170000	79.940000	95.000000	102.590000
	NS398						
-9.000000	6.680000	-2.370000	-1.360000	1.770000	-1.140000	-2.060000	0.930000
-8.000000	13.500000	-0.600000	-2.480000	8.280000	45.690000	11.930000	5.580000
-7.000000	47.150000	67.330000	74.960000	97.730000	96.820000	97.620000	87.570000
-6.000000	55.890000	69.740000	87.650000	100.770000	97.310000	97.840000	88.910000
	INDO						
-9.000000	3.940000	-1.780000	-3.200000	0.420000	1.310000	-0.620000	2.580000
-8.000000	7.410000	-0.680000	5.300000	17.130000	3.230000	25.570000	15.100000
-7.000000	40.780000	52.280000	75.730000	98.330000	96.800000	89.090000	58.860000
-6.000000	59.410000	86.790000	83.790000	99.890000	99.640000	96.430000	65.860000

Acetylcholine induced relaxation in the presence of DMSO. NS398 and indomethacin in Male D+

Acetylcholine doses [10^M/L]	DMSO						
-9.000000	-2.200000	-1.450000	0.240000	-1.940000	1.360000	-2.140000	1.050000
-8.000000	17.980000	15.720000	8.490000	3.850000	4.720000	-3.620000	6.660000
-7.000000	96.540000	89.890000	91.340000	94.180000	86.010000	85.400000	52.610000
-6.000000	97.850000	92.560000	94.650000	94.230000	95.660000	96.100000	71.890000
	NS398						
-9.000000	-3.990000	-4.030000	-1.480000	2.510000	1.460000	5.540000	-3.840000
-8.000000	6.900000	41.130000	-0.680000	54.730000	17.760000	18.250000	-2.880000
-7.000000	92.440000	50.620000	59.180000	95.940000	92.450000	85.580000	63.130000
-6.000000	98.930000	64.720000	79.550000	98.670000	99.550000	97.650000	84.690000
	INDO						
-9.000000	2.490000	0.360000	-0.520000	2.580000	0.120000	-3.570000	-2.800000
-8.000000	31.210000	15.630000	40.790000	35.160000	5.270000	1.520000	-2.610000
-7.000000	92.420000	85.830000	98.100000	99.700000	98.550000	94.240000	94.010000
-6.000000	96.130000	90.260000	103.800000	101.120000	99.940000	100.490000	99.660000

Acetylcholine induced relaxation in the presence of DMSO. NS398 and indomethacin in Male D-

Acetylcholine doses [10^M/L]							
	DMSO						
-9.000000	-2.680000	-3.520000	15.020000	0.270000	-7.990000	7.730000	-5.170000
-8.000000	-2.690000	-5.400000	5.930000	1.500000	-11.450000	9.410000	-9.330000
-7.000000	44.850000	66.300000	45.520000	16.350000	22.850000	75.960000	34.330000
-6.000000	86.310000	92.810000	75.880000	76.990000	90.070000	87.570000	87.960000
	NS398						
-9.000000	-2.230000	12.730000	-0.120000	0.920000	3.890000	15.390000	-1.120000
-8.000000	-3.770000	38.060000	0.160000	-0.090000	7.110000	15.120000	0.790000
-7.000000	87.220000	71.910000	57.190000	8.300000	75.310000	60.060000	17.790000
-6.000000	93.680000	81.830000	97.090000	96.600000	86.570000	85.290000	75.460000
	INDO						
-9.000000	-4.290000	-2.020000	-6.100000	-2.970000	-14.010000	1.370000	-0.360000
-8.000000	-2.430000	-5.520000	-10.270000	2.590000	-11.820000	9.170000	1.150000
-7.000000	94.190000	90.020000	68.330000	29.550000	92.140000	83.960000	49.580000
-6.000000	100.530000	97.940000	96.340000	96.290000	100.190000	99.260000	79.240000

Resorcin fuchsin straining. Intima/media area %

Female D+	Female D-	Male D+	Male D-
16.126110	23.553300	14.570470	21.766450
18.562040	12.644910	17.262050	17.211420
15.361280	16.304100	17.701350	15.121450
14.733660	18.094080	15.305280	17.635480
12.289530	14.147260		
	24.441570		

Resorcin fuchsine staining. Optical density

Female D+	Female D-	Male D+	Male D-
0.100845	0.174905	0.131891	0.077668
0.156230	0.200000	0.105852	0.142107
0.069149	0.247241	0.080764	0.102578
0.137727	0.163303	0.064261	0.085339
0.078720	0.162620		
	0.161557		

Smooth muscle actin IHC. Optical density

Female D+	Female D-	Male D+	Male D-
0.618492	0.784966	0.921854	0.722432
0.619533	0.569906	0.977626	0.834407
0.636233	0.630224	0.941693	0.743933
0.591534	0.558686	0.876943	0.887239
0.677254	0.713544		

eNOS IHC. Optical density

Female D+	Female D-	Male D+	Male D-
0.204447	0.159879	0.173255	0.182176
0.224750	0.168632	0.200270	0.199365
0.200648	0.186647	0.295601	0.247340
0.232344	0.161042		
0.302458	0.200334		

AT1R IHC. Optical density

Female D+	Female D-	Male D+	Male D-
0.173953	0.195982	0.163398	0.229818
0.158859	0.183296	0.188530	0.239882
0.207079	0.161899	0.215438	0.248703
0.204641	0.163670	0.262265	0.288400
0.153355	0.156303	0.182525	
	0.177646		

Number of smooth muscle cell nuclei in the media layer/1000µm^2

Female D+	Female D-	Male D+	Male D-
2.820147	5.156650	4.643318	3.309561
4.310325	2.371090	2.912600	4.619715
2.546857	3.669836	4.839090	4.071830
3.886765	3.352818	4.384508	4.677612
4.252644	2.668056		
